# Transcriptome analysis revealed the role of moderate exogenous methyl jasmonate treatments in enhancing the metabolic pathway of L-borneol in the *Blumea balsamifera*

**DOI:** 10.3389/fpls.2024.1391042

**Published:** 2024-06-26

**Authors:** Lingliang Guan, Na Lin, Lingyun Wan, Fulai Yu, Xiaolu Chen, Xiaoli Xie, Chao Yuan, Salma A. Soaud, Mohamed A. Abd Elhamid, Rania M. Y. Heakel, Linghui Wang, Ahmed H. El-Sappah

**Affiliations:** ^1^ Tropical Crops Genetic Resources Institute, Chinese Academy of Tropical Agricultural Sciences, Haikou, China; ^2^ Key Laboratory of Biology and Cultivation of Herb Medicine (Haikou), Ministry of Agriculture and Rural Affairs, Haikou, China; ^3^ School of Agriculture, Forestry and Food Engineering, Yibin University, Yibin, China; ^4^ Guangxi Key Laboratory of High-Quality Formation and Utilization of Dao-di Herbs, Guangxi Botanical Garden of Medicinal Plants, Nanning, China; ^5^ Genetics Department, Faculty of Agriculture, Zagazig University, Zagazig, Egypt

**Keywords:** Ainaxiang, cytoplasmic mevalonate, exogenous treatment, RNA sequencing, geranyl diphosphate

## Abstract

**Introduction:**

*Blumea balsamifera* L. (Ainaxiang) DC. is a perennial herb of the compositae family. It is also the primary source of natural borneol. Endo-borneol, the principal medical active element in *B. balsamifera*, is anti-inflammatory, antioxidant, and analgesic; enhances medicine absorption; refreshes; and is used as a spice and in cosmetic. Industrialization of *B. balsamifera* is limited by its low L-borneol concentration. Thus, understanding the accumulation pattern of the secondary metabolite endo-borneol and its synthesis process in secondary metabolism is critical for increasing *B. balsamifera* active ingredient content and cultivation efficiency.

**Methods:**

In this work, *B. balsamifera* was treated with varying concentrations (1.00 and 10.00 mmol/L) of methyl jasmonate (MeJA) as an exogenous foliar activator. The physiological parameters and L-borneol concentration were then assessed. Transcriptome sequencing of *B. balsamifera*-induced leaves was used to identify key genes for monoterpene synthesis.

**Results:**

The treatment effect of 1 mmol/L MeJA was the best, and the leaves of all three leaf positions accumulated the highest L-borneol after 120 h, correspondingly 3.043 mg·g^−1^ FW, 3.346 mg·g^−1^ FW, and 2.044 mg·g^−1^ FW, with significant differences from the control. The main assembly produced 509,285 transcripts with min and max lengths of 201 and 23,172, respectively. DEG analysis employing volcano blots revealed 593, 224, 612, 2,405, 1,353, and 921 upregulated genes and 4, 123, 573, 1,745, 766, and 763 downregulated genes in the treatments D1_1vsCK, D1_10vsCK, D2_1vsCK, D2_10vsCK, D5_1vsCK, and D5_10vsCK. Interestingly, when exposed to MeJA treatments, the MEP pathway’s unigenes express themselves more than those of the MVA route. Finally, when treated with 1 mmol/L, the genes *DXR*, *DXS*, and *GPS* showed increased expression over time. At the same time, a 10 mmol/L therapy resulted in elevated levels of *ispH* and *GGPS*.

**Discussion:**

Our preliminary research indicates that exogenous phytohormones can raise the level of L borneol in *B. balsamifera* (L.) DC when given in the appropriate amounts. The most significant discovery made while analyzing the effects of different hormones and concentrations on *B. balsamifera* (L.) DC was the effect of 1 mmol/L MeJA treatment.

## 1 Introduction

*Blumea balsamifera* (L.) DC, a half-woody plant that can easily grow in tropical and sub-tropical zones of Asia (Pang et al., 2014), belongs to the family *Asteraceae*, and the herbal medicine plant has been widely used for thousands of years in Southeast Asian countries, such as China, Philippines, Vietnam, and Thailand (Phumthum et al., 2018; Sadia et al., 2018; Lee et al., 2019). The most significant member of the *Blumea* genus, sambang, is an endemic plant to tropical and subtropical Asia, particularly China (Pang et al., 2014). This plant grows on the borders of woods, in river bottoms, valleys, and among grasslands. It is often a widely used plant in China, particularly in the provinces of Hainan, Guizhou, Yunnan, and Guangdong, and in Taiwan (Pang et al., 2010; Guan et al., 2012; Pang et al., 2014) and the Li-nationality People take baths in boiled leaves to improve various women’s problems, especially postpartum (Shirota et al., 2011). People frequently refer to *B. balsamifera* as “Ainaxiang” and “Dafeng’ai” in Chinese because of its high concentration of essential oils, and they burn it as incense (Pang et al., 2014). It was first written down in “Bei Ji Qian Jin Yao Fang” by Sun Simiao in the year 652 (Pang et al., 2014). Primitive Chinese traditional medicine uses the whole plant or its leaves to cure eczema, dermatitis, beriberi, lumbago, menorrhagia, rheumatism, skin injuries, and insecticides (Chen et al., 2010). Plants are the source of traditional Chinese medicines (TCMs), such as Bing Pian and Aipian, which are identical in potency and primarily contain borneol (Pang et al., 2014). In today’s Chinese pharmaceutical market, they are interchangeable. Before 2010, Sambong was one of Bing Pian’s most important plant sources. But since 2010, the Pharmacopoeia of the People’s Republic of China lists *B. balsamifera* as the only plant source for Aipian (Pang et al., 2014), with *B. balsamifera* medicinal materials that could bring back to life, clear heat, and ease pain. Recent studies (Nessa et al., 2004; Hasegawa et al., 2006; Kubota et al., 2009; Pang et al., 2014) have confirmed the anticancer, antifungal, radical scavenging, and anti-obesity effects of extracts of its leaves. In Chinese medicine, the decoction of leaves taken as an expectorant is common and can also be found in Malaysia, India, and Mizoram (Pang et al., 2014). In Vietnam, the vapor from boiling sambong leaves can be used as an inhalation for the treatment of cough and influenza (Ahmad, 2021). Recently, research has shown that *B. balsamifera* DC has new applications as a diuretic or in the dissolution of renal stones (Widhiantara and Jawi, 2021). A phytochemical investigation of *B. balsamifera* DC revealed that it contained volatile oil, and flavonoids (Pang et al., 2014; Masyudi et al., 2022). A total of 28 compounds were identified from the main essential oils, and the main forms are L-borneol and L-camphor (Masyudi et al., 2022). Borneol is a terpene derivative and exists as two enantiomers for chiral. Both D-borneol and L-borneol are found in nature. L-Borneol is the base of the volatile oil from *B. balsamifera* (L.) DC (Wang et al., 2014). Pharmacological studies show L-borneol has activities of inhibiting bacteria, antioxidants, anti-inflammatory, and analgesia, and also contributes to pharmaceutical absorption and deodorizing competitiveness (Moghtader et al., 2011; Ma et al., 2023). L-Borneol is a secondary metabolite of *B. balsamifera* (L.) DC and its active pharmaceutical ingredients (Bhuiyan et al., 2009; Widhiantara and Jawi, 2021).

*B. balsamifera* DC has grown abundantly for medicine and cosmetics recently (Pang et al., 2014; Jirakitticharoen et al., 2022). The metabolite accumulation pattern of *B. balsamifera* DC could be affected by cultivating conditions and the external environment (Wang et al., 2023). Research revealed that the content of L-borneol was different in different tissues at different developmental stages (Pang et al., 2017; Guan et al., 2022). Mei et al. (2023) observed that tender leaves have the highest L-borneol compared with other tissues of *B. balsamifera*. Much research focuses on accumulating more secondary metabolites in medicinal plants (Al-Gabbiesh et al., 2015; Pant et al., 2021). Jasmonic acid (JA) and its derivative are widely distributed in plants as signal molecules and can also be inducers to regulate the biosynthesis of metabolites (Turner et al., 2002; Ruan et al., 2019). The preliminary study of our group demonstrated that the treatment of exogenous phytohormones with a suitable concentration range can improve the content of L-borneol in *B. balsamifera* (L.) DC, with the effect of 1 mmol/L methyl jasmonate (MeJA) treatment on *B. balsamifera* (L.) DC being the most outstanding when compared to other hormones and other concentrations. To understand the accumulation mechanisms of L-borneolin *B. balsamifera* (L.) DC with the MeJA treatment, we screen the key regulation gene, identify its function, and detect the transcriptome and metabolome.

## 2 Materials and methods

### 2.1 Experimental materials and exogenous hormone treatments

Individual *B. balsamifera* plants were selected for vegetative propagation at the germplasm repository of the tropical medicinal plant greenhouse (19°30′N, 109°20′E; temperature, 28°C; humidity, 60%; light application time, 9 h) of Ministry of Agriculture in Danzhou city, Hainan province, which is linked with the Tropical Crops Genetic Resources Institute, Chinese Academy of Tropical Agricultural Sciences. The nursery garden has red clay soil, partly sandy. Every plant was grown for 3 months. The identical plants were separated into five groups based on leaf count and phenotype, each with 20 plants. Each MeJA solution was produced in 1 L of ddH_2_O, which provided sufficient volume for foliar applications. Each plant was continuously sprayed with 20 mL of a surface-treating chemical. In addition to the control (distal water), each foliar treatment received equal exposure to MeJA doses of 1 mmol/L and 10 mmol/L. A volume of 20 ml of surface-treating chemicals were added to the spray bottle and equably sprayed at 20 cm from the plants. Each treatment was given three times in total. After the treatment, mature leaves were gathered at 24 h, 48 h, and 120 h. The samples were immediately stored in liquid nitrogen and kept at −80°C, until they were used for qPCR, RNA sequencing (RNA-seq), and metabolite analysis.

### 2.2 Determination of L-borneol

The collected *B. balsamifera* leaves were immediately placed in a mortar and ground with liquid nitrogen, and ground into a powder. We accurately weighed 2 g of the ground powder into a 50-mL centrifuge tube and added 25 mL of ethyl acetate, operating at 40 kHz and 400 W. We performed an ultrasonic extraction for 30 min, left it standing, and filtered it. Add 1 mL of the filtrate to a 10-mL volumetric flask, along with 1 mL of methyl salicylate internal standard solution. Next, fix the volume with ethyl acetate and filter through a 0.22-μm microporous filter membrane. The content of L-borneol was determined by GC-MS, HP-5 quartz capillary column (0.32 mm × 30 m, 0.25 μm), starting at 80°C for 2 min, then heating at 5°C/min to 100°C, 20°C/min to 200°C. The temperatures of the inlet and FD detector were 220°C and 240°C, respectively. The injection volume is 0.6 μL, without shunt.

### 2.3 RNA isolation, library construction, and transcriptome sequencing

TRIzol reagent (Invitrogen) was used to extract total RNA, and the manufacturer’s instructions were followed (El-Sappah et al., 2023). Using the NanoPhotometer1 spectrophotometer (IMPLEN, CA, USA) and the Qubit1 RNA Assay Kit in Qubit1 2.0 Flurometer (Life Technologies, CA, USA), total RNA purity and concentration were assessed. RNA integrity was evaluated using the Agilent Bioanalyzer 2100 system (Agilent Technologies, CA, USA) and the RNA Nano 6000 Assay Kit. The RNA sample preparations employed a total of 1.5 g of RNA as input material for each sample. Utilizing the NEBNext1 UltraTM RNA Library Prep Kit for Illumina1 (NEB, USA) in accordance with the manufacturer’s instructions, sequencing libraries were created, and index codes were applied to assign sequences to specific samples. The TruSeq PE Cluster Kit v3-cBot-HS (Illumina) was used on the acBot Cluster Generation System to cluster the index coded sample data in accordance with the manufacturer’s recommendations. Using an Illumina Hiseq 4000 platform, the library preparations were sequenced after cluster creation, and paired-end reads were produced. Fastq format raw readings were processed using internal perl scripts as part of the quality control stage. By eliminating adapter- and ploy-N-containing reads and low-quality reads from the raw data in this stage, clean reads were produced. At the same time, it was determined what the clean data’s Q20, Q30, GC content, and sequence duplication level were. On clean, high-quality data, all subsequent analyses were performed. During the transcriptome assembly process, all libraries’ and samples’ left files (read1 files) were merged into a single big left.fq file, and all libraries’ and samples’ right files (read2 files) into a single large right.fq file. Using Trinity (Grabherr et al., 2011), transcriptome assembly was completed with min_kmer_cov and all other parameters set to their default settings. Right.fq and left.fq were used as the foundation for this. Gene function was conducted as described before. The alignments of unigenes to the Nt database were carried out using NCBI blast 2.2.28C with an E-value threshold of 1E−5. The program diamond (v0.8.22) was used for the comparison with the Nr (E-value, 1E−5), KOG/COG (E-value, 1E−3), and SwissProt (E-value, 1E−5) databases. Using an E-value limit of 0.01 and hmmscan from HMMER 3.0, Pfam was searched. With an E-value threshold of 1E−6 based on the Nr and Pfam annotations, the GO (Gene Ontology) annotations were carried out in Blast2GO (v2.5) (Wan et al., 2024). Pathway analysis was carried out to ascertain the crucial pathways of DEGs using the databases of the Kyoto Encyclopedia of Gene and Genomes (KEGG) (http://www.genome.jp/kegg). The statistical enrichment of differentially expressed genes in KEGG pathways was evaluated using the KOBAS program. Genes that exhibit substantial levels of differential expression are found in the pathways with an FDR value of 0.05 (Wan et al., 2023).

### 2.4 Quantitative real-time PCR validation

Five DEGs were chosen for quantitative real-time PCR (qRT-PCR) to validate the transcriptome results. All cDNAs were generated using the Prime Script RT reagent Kit with gDNA Eraser (TaKaRa, Kyoto, Japan), and qRT-PCR experiments were performed using the ABI 7500 Fast Real-Time Detection System and the TaKaRa SYBR Green Mix kit (TaKaRa, Kyoto, Japan). According to Li et al. (2021), the amplification system and process were used. The reference genes were Primer Premier 5.0-designed primers and 18S rRNA. **Supplementary Table S1** lists all of the primers utilized. The 2CT approach was used to calculate the relative expression level.

### 2.5 Statistical analysis

The data were examined using Microsoft Excel 2007. The Statistical Analysis System v. 9.2 was used for significance testing (Duncan’s test) and principal component analysis (PCA). Heatmaps and Venn diagrams were drawn using Tbtools, while other figures were drawn using Origin 2022.

## 3 Result

### 3.1 L-Borneol concentration under different MeJA treatments

**Figure 1:**
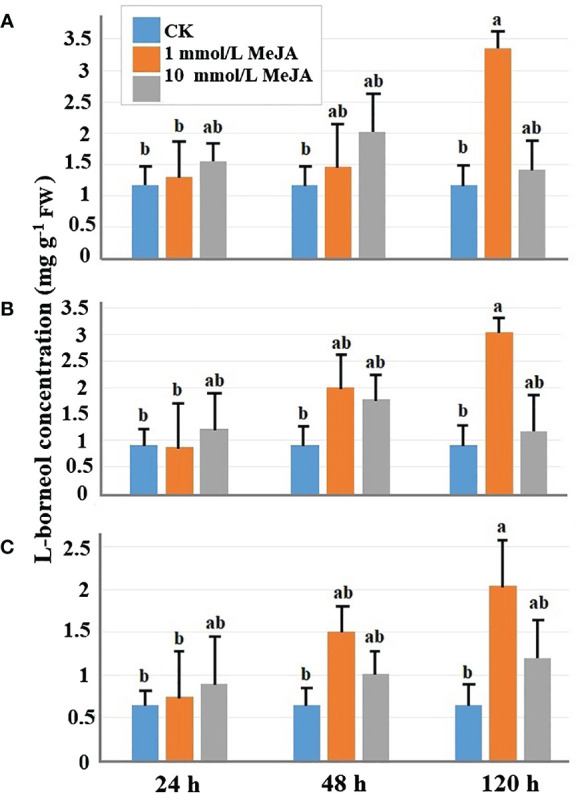
The L-borneol concentration of *B. balsamifera* mature leaves at three different times (24 h, 48 h, and 120 h) under different MeJA treatments: **(A)** the young leaves, **(B)** mature leaves, and **(C)** old leaves. The P <0.05 was denoted by the lowercase letters.

Different concentrations of MeJA affect the accumulation of L-borneol. Overall, different concentrations of MeJA can both promote L-borneol accumulation in the plant’s leaves. Except for the 10 mmol/L MeJA of 120-h treatment, moxa under other concentration treatments, there is accumulation of L-borneol in the leaves of the three leaf positions of Ainaxiang over time, especially when the concentration was 1 mmol/L on 48-h treatment. The treatment effect of 1 mmol/L MeJA was the most effective, resulting in the highest accumulation of L-borneol at 120 h in leaves at different stages of maturity (young, mature, and old leaves), with concentrations of 3.043 mg·g^−1^ FW, 3.346 mg·g^−1^ FW, and 2.044 mg·g^−1^ FW, respectively. These values showed significant differences compared to the control. **Figure 1** clearly demonstrates the positive impact of 1 mmol/L MeJA treatment on the accumulation of L-borneol in the leaves of Ainaxiang.

### 3.2 Sequencing and differentially expressed genes analysis of transcriptomes

To explore MeJA’s regulation mechanism on terpenoid synthesis in the leaves of Ainaxiang, non-induced and induced conditions were selected. To obtain the transcriptome, Illumina high-throughput next-generation sequencing was performed on the mature leaves of Ainaxiang under different conditions. The clean data screening requirements are tightly regulated in order to assure the caliber of the subsequent analysis. The reads with adapters should be eliminated first, and then, the reads that make up more than 10% but whose base information cannot be identified. The gathered clean reads for the non-reference genome were spliced using Trinity before the sequence was examined. The primary assembly yielded a total of 509,285 transcripts with minimum and maximum lengths of 201 and 23,172, respectively, and most of them (280,106) are between 200 bp and 500 bp, followed by those in the 501–1,000 bp range (82,683 transcripts) (**Supplementary Table S2**). In addition, 68,679 transcripts were observed in the range of 1,001–2,000 bp, and 77,817 transcripts were in the range of more than 2,000 bp, with average transcript length of 989 bp and N50 of 2,088 bp.

### 3.3 Gene function annotation and classification

**Figure 2:**
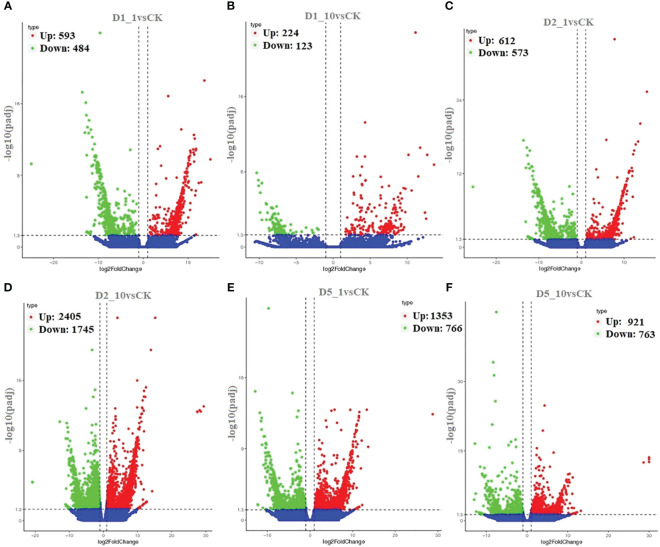
Identification of differentially expressed genes (DEG). Volcano plot showing the differentially expressed genes of **(A)** D1_1vsCK, **(B)** D1_10vsCK, **(C)** D2_1vsCK, **(D)** D2_10vsCK, **(E)** D5_1vsCK, and **(F)** D5_10vsCK.

**Figure 3:**
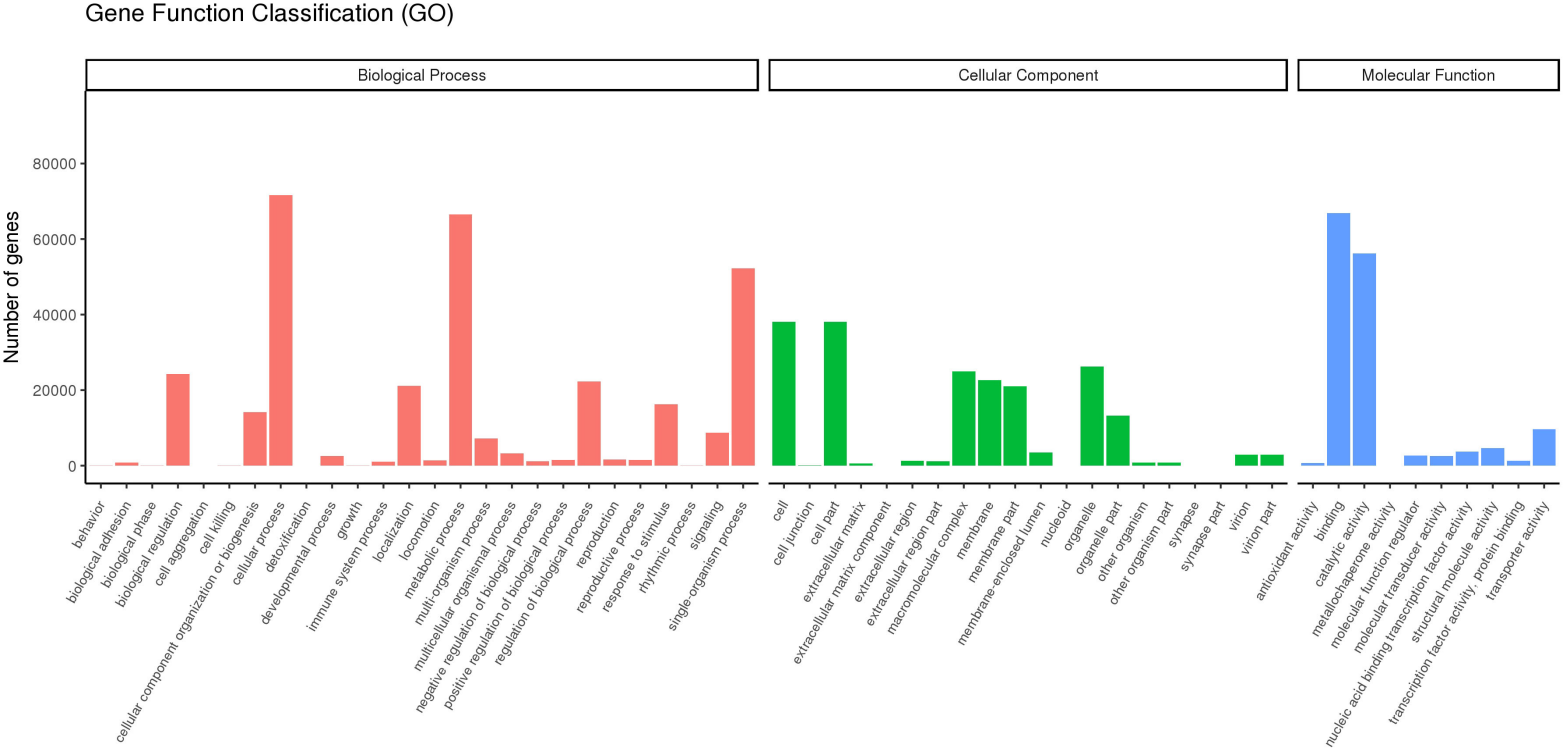
Unigene classification based on gene ontology (GO). These unigenes were classified into three groups based on functional annotation: biological process, cellular component, and molecular function.

We aligned the 267,178 unigenes to the Nr (NCBI non-redundant protein sequence), Nt (NCBI non-redundant nucleotide sequence), Pfam (protein family), KOG (protein ortholog source clusters), Swiss-Prot (manually annotated and reviewed protein sequence database), and KO (KEGG Ortholog database), achieving a match of 23.47% × 65.36, for example. In **Figure 2**, comparing DEG using a volcano blot showed that 593, 224, 612, 2,405, 1,353, and 921 genes were upregulated, and 4, 123, 573, 1,745, 766, and 763 genes were downregulated in the D1_1vsCK, D1_10vsCK, D2_1vsCK, D2_10vsCK, D5_1vsCK, and D5_10vsCK treatments, in that order. We used GO enrichment analysis to anticipate the activities of the DEGs. We found that the DEGs enriched for three primary ontologies: biological process, cellular component, and molecular function. We assigned the 666,068 unigenes to three major GO terms: cellular component (198,296 unigenes across all samples), molecular function (148,057 unigenes), and biological process (319,733 unigenes). Finally, we annotated the 62,720 DEGs of all samples to 25 GO terms, including posttranslational modification, protein turnover, chaperones category, which contained 6,248 unigenes, and translation, which had 2383 unigenes related (**Figure 3**; **Supplementary Table S3**).

**Figure 4:**
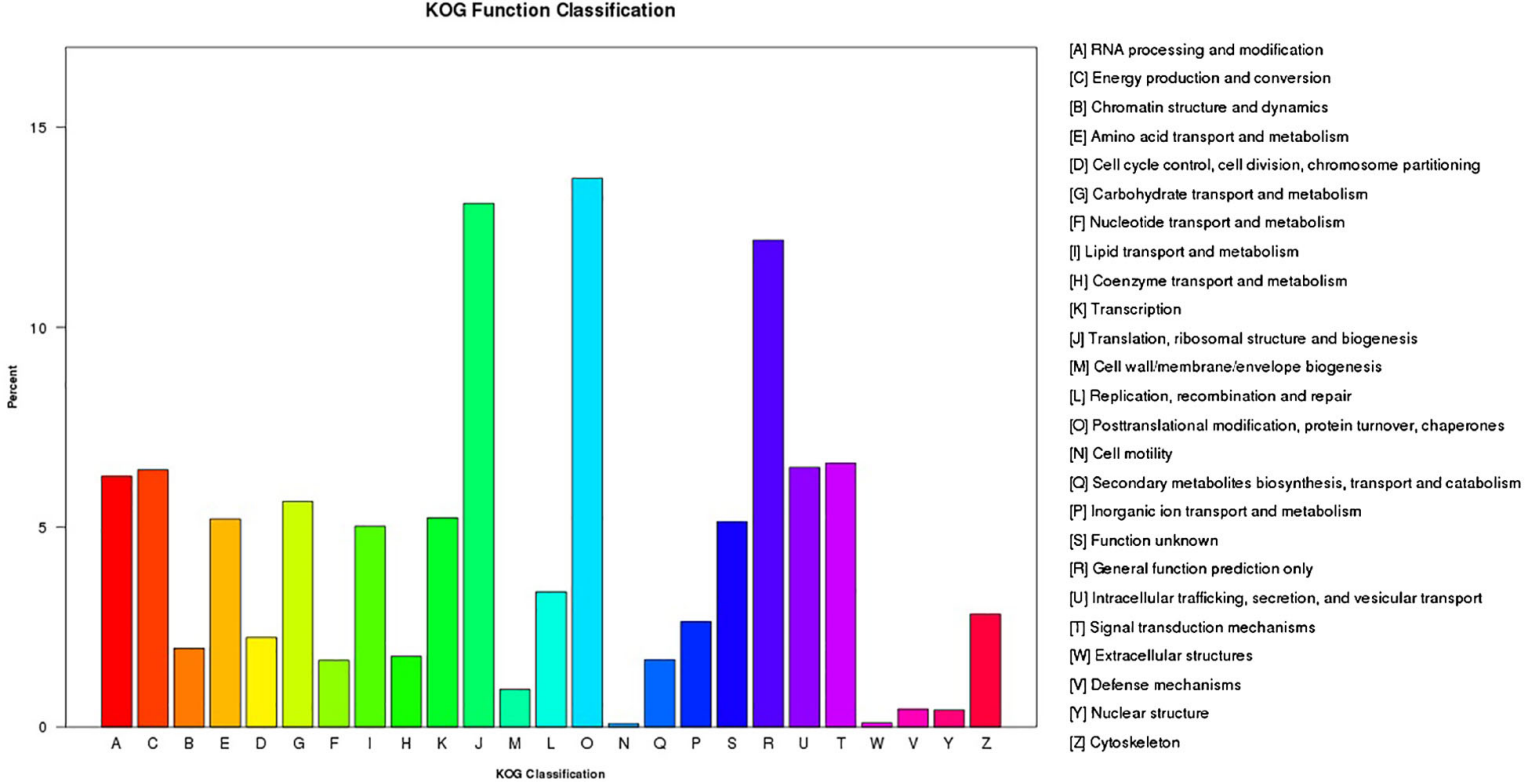
Unigene classification by eukaryotic orthologous groups (KOG). According to the KOG database, functional predictions of unigenes were grouped into at least 25 function classes.

**Figure 5:**
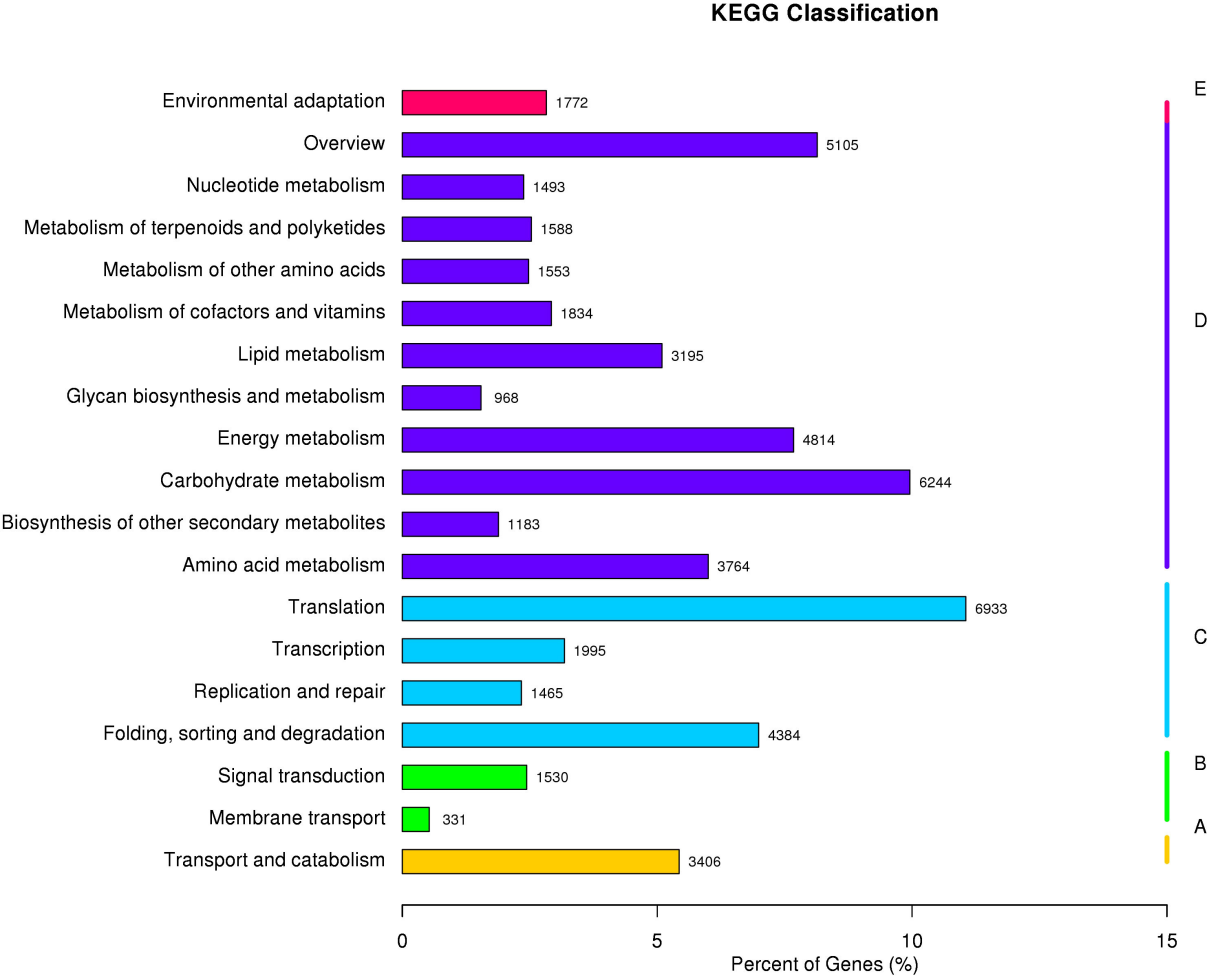
Unigene metabolic pathway classification according to the Kyoto Encyclopedia of Genes and Genomes (KEGG). The data were divided into five categories: **(A)** cellular processes, **(B)** environmental information processing, **(C)** genetic information processing, **(D)** metabolism, and **(E)** organismal systems. The number of *B. balsamifera* unigene matches in each category is represented by the bars.

In addition, the KOG database showed that 50,632 unigenes had strong functional prediction and categorization matches (**Figure 4**; **Supplementary Table S4**). The 25 KOG categories that were looked at were translation, ribosome structure and biogenesis (5963), general function prediction alone (5544), and signal transduction pathways (3005). The largest groups were posttranslational modification, protein turnover, and chaperones (6248). The smallest categories were extracellular structures (46) and cell motility (40). We also examined the unigenes’ KEGG pathways. We found that 25 projected metabolic pathways (**Figure 5**; **Supplementary Table S5**), grouped into five categories: metabolism, genetic information processing, environmental information processing, cellular activities, and organismal systems, involved 64803 unigenes. The most numerous category in its gene content was metabolism, including carbohydrate metabolism (6244), followed by overview (5105), energy metabolism (4814), amino acid metabolism (3764), and lipid metabolism (3195). As demonstrated in **Figure 5** and **Supplementary Table S5**, the smallest categories were organismal systems (1772) and environmental information processing (1861).

### 3.4 Analysis of DEGs involved in the (−)-borneol biosynthesis pathway

**Figure 6:**
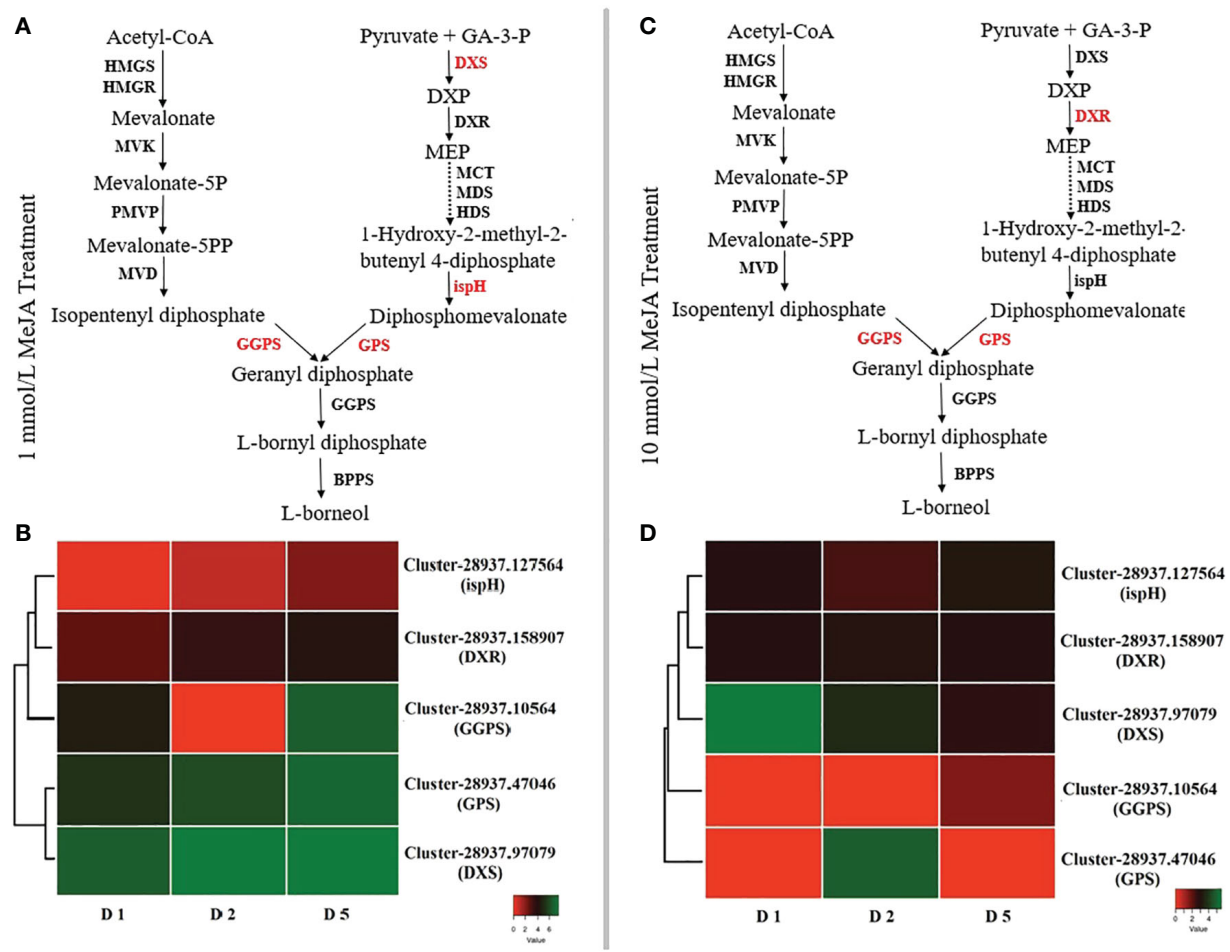
DEGs responsible for the (–)-borneol biosynthesis pathway in *B. balsamifera*: **(A)** the borneol biosynthesis pathway at 1 mmol/L MeJA; **(B)** a heat map of genes involved in borneol biosynthesis at 1 mmol/L MeJA; **(C)** the borneol biosynthesis pathway at 10 mmol/L MeJA; and **(D)** a heat map of genes involved in borneol biosynthesis at 10 mmol/L MeJA. D1 (Day 1) represents 24 hours, D2 48 hours, and D5 120 hours. Every pathway’s red-colored genes indicate an upregulated expression level.

The expression profiles of genes involved in borneol production were studied to investigate the regulatory mechanisms for the accumulation patterns of diverse borneol in *B. balsamifera* under different MeJA treatments. *B. balsamifera* was found to include 4,848 expressed unigenes that encode terpenoid biosynthesis enzymes. At 24 h after 1 mmol/L MeJA treatment, there are 1,077 DEGs, 1,185 DEGs at 48 h, and 2,119 DEGs at 120 h. At each sample point, there were 593, 484, and 612 upregulated genes. They also had 347, 4,150, and 1,684 DEGs at each point under 10 mmol/L MeJA treatment; upregulation genes were 224, 123, and 2,405 at three sample points, respectively. **Figure 6** displays the FPKM values and expression data for each unigene. Most genes that code for important enzymes in the borneol backbone pathway (MEP and MVA pathways, KEGG entry ko00900) did have a high transcriptome expression level. However, the deoxy-d-xylulose 5-phosphate synthase (*DXS*), *ispH*, geranylgeranyl diphosphate synthase (*GGPS*), geranyl diphosphate synthase (*GPS*), and 1-deoxy-d-xylulose-5-phosphate reductoisomerase (*DXR*) unigenes did not. Under the low concentration of MeJA treatment, *DXS* (Cluster-28937.97079) exhibits high expression on all treatment days. At the same time, every *ispH* (Cluster-28937.127564), *GGPS* (Cluster-28937.10564), and *GPS* (Cluster-28937.47046) showed upregulation only after 120 h treatment. On the other hand, under 10 mmol/L MeJA treatment, the expression of *GGPS* (Cluster-28937.10564) and *GPS* (Cluster-28937.47046) showed upregulation in all treatment days, and *DXR* (Cluster-28937.158907) showed upregulation at the first 48 h, while *DXS* (Cluster-28937.97079) exhibited a high expression level after 120 h of treatment. Interestingly, unigenes in the MEP route have greater expression levels than those in the MVA pathway.

### 3.5 Gene expression of the L-borneol biosynthesis pathway related genes

**Figure 7:**
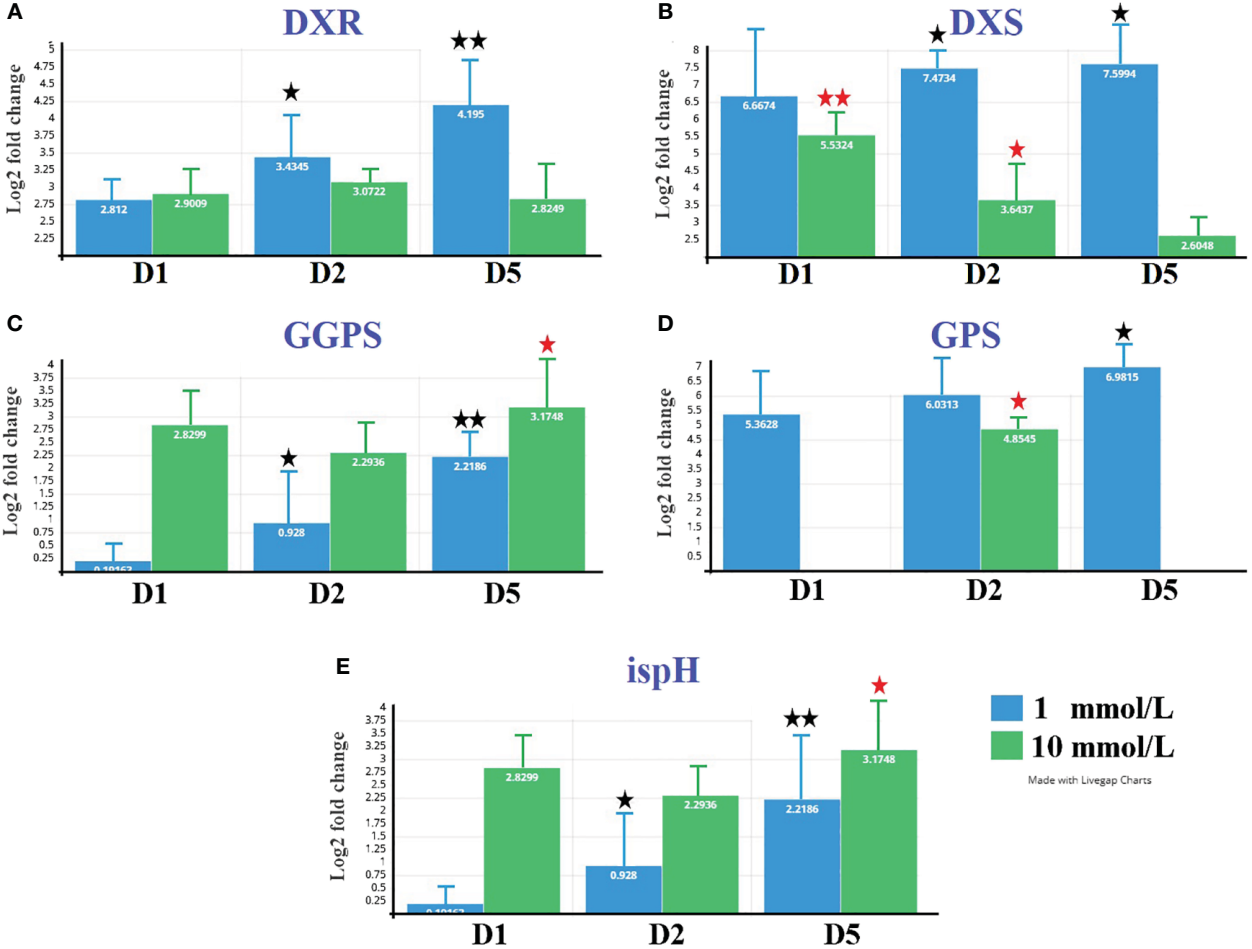
Expression profile of five selected unigenes related to (−)-borneol biosynthesis pathway in *B. balsamifera* under both MeJA concentrations. Unigene expression was analyzed by qRT-PCR using *Actin* gene as an internal reference. Data represent means ± standard deviation (SD) from three biological replicates (three technical replicates per biological replicate). Repetition of the experiment produced similar results. * Different than pre (P < 0.05).

The expression analysis related to borneol biosynthesis genes was conducted at both 1 and 10 mmol/L MeJA treatments, as shown in **Figure 7**. The gene expression results are consistent with the transcriptomic analysis results. Genes *DXR*, *DXS*, and *GPS* showed high expression over time at 1 mmol/L treatment. At the same time, *ispH* and *GGPS* showed high expression with a 10 mmol/L treatment.

## 4 Discussion

L-Borneol, which has a high degree of volatility, is the major active substance. Furthermore, researchers discovered a variety of biological actions in essential oils, flavonoids, and terpenoids (Chen et al., 2009). These investigations may help to understand the many pharmacological actions of this plant.

The MeJA, is an important cellular regulator that controls how plants grow and defend themselves in response to both living and non-living factors (Cheong and Do Choi, 2003; Wolucka et al., 2005). MeJA, when used before harvest, is considered safe for all food products (García-Pastor et al., 2020; Dhiman et al., 2022). The major technique for raising the number of secondary metabolites in vegetables is phytohormone induction, which results in a reaction to stress in plants (Sharma et al., 2022). The most extensively researched and applied excitons for promoting the synthesis of secondary metabolites in agricultural products to improve their nutritional quality are JA and MeJA (Wang et al., 2022). Exogenous MeJA has the ability to modify the concentrations of several primary metabolites, such as plant sugars, organic acids, and amino acids (Kim et al., 2017; Meza et al., 2022). *Brassica oleracea* L. var. italica (Plenck) treatment with MeJA promotes the production of bioactive chemicals (Ilahy et al., 2020; Bao et al., 2022). Treatment with MeJA also encourages the synthesis of volatile molecules, the buildup of non-volatile secondary metabolites, and secondary metabolites with a longer half-life (Bourgougnon and Stiger-Pouvreau, 2011; Singh et al., 2016). The production of aromatic compounds and the buildup of β-carotene are both stimulated by exogenous JA (Fan et al., 1998; Wang et al., 2022). MeJA, according to Liu et al. (2012) enhanced the expression of genes essential for carotenoid production, which in turn increased the accumulation of lycopene in tomato (*Lycopersicon esculentum*) tissue. According to Shafiq et al. (2013), MeJA enhanced the formation of phenolic components in the pericarp of apples (*Malus pumila* Mill.), such as chlorogenic acid, anthocyanin 3-galactoside, and flavonols. Scientific studies have demonstrated a decreased incidence of chronic and degenerative illnesses, including cancer, with a diet high in naturally occurring polyphenols produced by plants (Pandey and Rizvi, 2009; Zhang and Tsao, 2016). This is mostly due to the fact that phenolic compounds rich in phenolic acids, anthocyanins, and flavonoids usually exhibit potent free radical scavenging properties (Weng and Yen, 2012). When exposed to MeJA, the bioactive components of blackberry (*Rubus fruticosus* Pollich) and pomegranate (*Punica granatum* L.) show increased antioxidant and other positive effects (Wang et al., 2008; Sayyari et al., 2011). In the present study, the plants of *B. balsamifera* grown for 3 months were used as materials, different concentrations (1.00 mmol/L and 10.00 mmol/L) of MeJA were exogenous activators, their physiological indexes and L-borneol were determined, and the optimal concentration and time were screened in *B. balsamifera* induction. To identify the crucial genes in the manufacture of monoterpene, the transcriptome of the induced leaves of *B. balsamifera* was sequenced. The main source of (−)-borneol is the initial geranyl diphosphate (GPP), which is made from the two common C5 building blocks isopentenyl diphosphate and dimethylallyl diphosphate using either the cytoplasmic mevalonate (MVA) or the plastidic 2-C-methyl-D-erythritol-4-phosphate (MEP) pathways (Lichtenthaler, 1999; Rehman et al., 2016; Tian et al., 2022). Numerous (+)-bornyl diphosphate synthases have been identified in plants, including *Cinnamomum burmanni*, *Salvia officinalis*, *Amomum villosum*, *Lippia dulcis*, and *Lavandula angustifolia*, but it is still unclear which monoterpene synthase is responsible for cycling GPP to form (−)-bornyl diphosphate (Chen et al., 2023). The analysis of the gene expression profiles for those involved in the production of borneol revealed that 1 mmol/L MeJA therapy had a positive impact on the accumulation of L-borneol.

*B. balsamifera* leaves exhibited a total of 4,848 expressed unigenes that encode terpenoid biosynthesis enzymes. With the exception of the deoxy-d-xylulose 5-phosphate synthase (*DXS*), *ispH*, *GGPS*, *GPS*, and *DXR* unigenes, the majority of the genes encoding important enzymes in the borneol backbone pathway (MEP and MVA pathways, KEGG entry ko00900) had a high transcriptome expression level. Furthermore, under the low concentration of MeJA treatment, the expression of *DXS* (Cluster-28937.97079) exhibits high expression at all treatment days, while every expression of *ispH* (Cluster-28937.127564), *GGPS* (Cluster-28937.10564) and *GPS* (Cluster-28937.47046) showed upregulation only after 120 h treatment. Previous studies revealed that the 1-deoxy-D-xylulose-5-phosphate synthase (*DXS*) and two monoterpene synthases, known as CbDXS9, CbTPS2, and CbTPS3, were upregulated in the high-borneol group compared to the low-borneol and borneol-free groups and may be crucial for the biosynthesis of D-borneol in *C. burmannii* (Yang et al., 2020). *Arabidopsis IspH* gene expression in both photosynthetic and nonphotosynthetic tissues lends credence to the idea that the non-MVA pathway is involved in the synthesis of a range of isoprenoids in plants (Hsieh and Goodman, 2005). The enzyme known as geranylgeranyl diphosphate synthase (GGPPS) is essential for diterpene biosynthesis because it catalyzes the synthesis of GGPP, a frequent precursor to diterpenes (Zhang et al., 2015). Additionally, GPPS catalyzes the condensation of isopentenyl diphosphate and dimethylallyl diphosphate to yield geranyl diphosphate (Gilg et al., 2005). Several earlier studies found a strong link between the *DXR* gene’s activity and the amount of essential oil in *C. camphora* (Hou et al., 2020). This suggests that the upregulated *DXR* gene expression in *C. camphora* could lead to an increase in terpenoid content (Du et al., 2022). Wang et al. (2023) indicated that the *OfDXR* gene is very important for making terpenoid compounds and that overexpressing it in *Arabidopsis* plants during the main or full blooming stage led to a rise in the total amount of terpenoid compounds. Zhang et al. (2022) found that *DXS*, *DXR*, and *ispH* in the MEP pathway and GGPS were significantly expressed in summer Chinese cedar (SCC) needles and increased the accumulation of terpenoids, notably diterpenoids.

## Data availability statement

The original contributions presented in the study are publicly available. This data can be found at the National Center for Biotechnology Information (NCBI) using accession number PRJNA668407, as well as in the article/**Supplementary Material**.

## Ethics statement

This article does not contain any studies with human participants or animals performed by any of the authors.

## Author contributions

LG: Conceptualization, Data curation, Formal analysis, Funding acquisition, Investigation, Methodology, Project administration, Resources, Visualization, Writing – original draft, Writing – review & editing. NL: Data curation, Formal analysis, Methodology, Resources, Writing – review & editing. LYW: Investigation, Software, Validation, Visualization, Writing – review & editing. FY: Formal analysis, Methodology, Resources, Visualization, Writing – review & editing. XC: Data curation, Formal analysis, Methodology, Writing – review & editing. XX: Methodology, Resources, Software, Writing – review & editing. CY: Formal analysis, Investigation, Methodology, Writing – review & editing. LHW: Data curation, Investigation, Writing – review & editing. SS: Data curation, Formal analysis, Writing – review & editing. MA: Data curation, Formal analysis, Writing – review & editing. RH: Data curation, Formal analysis, Writing – review & editing. AE: Conceptualization, Data curation, Formal analysis, Methodology, Validation, Writing – original draft, Writing – review & editing.
