# Supplementary material for: Transcriptome analysis revealed the role of moderate exogenous methyl jasmonate treatments in enhancing the metabolic pathway of L-borneol in the Blumea balsamifera
Source: Front Plant Sci. 2024 Jun 26;15:1391042. doi: 10.3389/fpls.2024.1391042 (PMC11234090; doi:10.3389/fpls.2024.1391042)
Supplement: Supplementary file 1 [file Table_1.docx]

Table S1 List of primers used in real time PCR analysis

| Gene | Primer |
| --- | --- |
| Cluster-28937.158907（DXR） | PF:TAATAGAATTTATCATACGG  PR:CTACAAGTGATAATCACATAC |
| Cluster-28937.97079 (DXS) | PF:TTGTGATTCCGAGTGCCGGT  PR:CAAGAGGCTTGCAGAATCTT |
| Cluster-28937.10564 (GGPS) | PF:GATTCTTCTTCCCAGTCGTTG  PR:GTCAATATCTCATTGACGGCAG |
| Cluster-28937.47046 (GPS) | PF:CAATTGTCCT ACTCCAGTCG  PR:GACAGGTAAG ATTTGTAAAC |
| Cluster-28937.127564 (ispH) | PF: CATCTTTCTCTCGGCAGAAC  PR: GTGTACATAGCTGCATGCATG |
